# Fernandes Figueira e a higiene infantil no Rio de Janeiro, 1880-1930

**DOI:** 10.1590/S0104-59702024000100002

**Published:** 2024-04-05

**Authors:** Gisele Sanglard, Letícia Conde Moraes Cosati

**Affiliations:** i Pesquisadora, Departamento de Pesquisa/Casa de Oswaldo Cruz/Fiocruz. Rio de Janeiro – RJ – Brasil gisele.sanglard@fiocruz.br; ii Doutoranda, Programa de Pós-graduação em História das Ciências e da Saúde/Casa de Oswaldo Cruz/Fiocruz. Rio de Janeiro – RJ – Brasil leticiacosati@gmail.com

**Keywords:** Children’s health, Antônio Fernandes Figueira (1863-1928, Rio de Janeiro, Higiene infantil, Antônio Fernandes Figueira (1863-1928, Rio de Janeiro

## Abstract

O artigo discute a relação entre o médico Antônio Fernandes Figueira e a higiene infantil no período de institucionalização dos estudos acerca da saúde da criança no Brasil. A higiene infantil é fruto da emergência do conceito de saúde em contraposição à doença. Interessa-nos compreender como a higiene infantil se estabeleceu, se em parceria ou oposição à pediatria. Enfatizamos como a circulação das ideias sobre a saúde da criança chegou ao país e como esse médico dialoga com as discussões europeias e as reinterpreta. Nosso fio condutor é a alimentação infantil como pilar da higiene infantil. Para tal, nos baseamos no Livro das mães: consultas práticas de higiene infantil, publicado pelo médico em 1910.

## Abstract (en)

This article discusses the relationship between the physician Antônio Fernandes Figueira and children’s health during the time that studies in this area were being institutionalized in Brazil. Children’s health resulted from the emergence of the concept of health in contrast with disease; here we focus on how children’s health became established, in partnership with or opposition to pediatrics. We emphasize how the circulation of ideas on children’s health reached Brazil, and how this physician dialogued with and reinterpreted European discussions. A central theme is children’s nutrition. We focused on Livro das mães: consultas práticas de higiene infantil [The mothers’ book: practical queries on children’s health], published by Fernandes Figueira in 1910.

Este artigo discute a relação entre o médico Antônio Fernandes Figueira (1863-1928) e a higiene infantil no período de institucionalização dos estudos acerca da saúde da criança no Brasil. Entende-se a higiene infantil como fruto da emergência do conceito de saúde em contraposição com a doença que é, por sua vez, fruto do higienismo do século XIX. Assim, interessa-nos compreender como a higiene infantil se estabeleceu, se em parceria ou oposição à pediatria, no momento de criação da cátedra na Faculdade de Medicina do Rio de Janeiro (FMRJ), em 1883, e no processo de formação profissional desse médico. Enfatizamos como a circulação das ideias sobre a saúde da criança chegou ao país e como esse médico dialoga e reinterpreta as discussões europeias.

Mais preocupado com a “prevenção” do que com o combate à doença, a ação médica de Fernandes Figueira será marcada pela “prevenção à mortalidade infantil”, sobretudo por meio da boa alimentação a ser ofertada à primeira infância – notadamente ao filho da classe trabalhadora; além de ser o responsável pelas primeiras políticas públicas voltadas para a infância.

O recorte temporal deste artigo refere-se à criação da cátedra de pediatria na FMRJ e na Faculdade de Medicina da Bahia, em 1883, fruto da reforma do ensino médico, e ao falecimento de Antônio Fernandes Figueira, em 1928. Além de ser considerado um dos principais nomes da primeira geração de pediatras, foi fundador da Sociedade Brasileira de Pediatria, em 1909, e seu presidente perpétuo, organizou, em 1923, a Inspetoria de Higiene Infantil (IHI), ligada ao Departamento Nacional de Saúde Pública (DNSP), além de ser um grande defensor da higiene infantil.

A baliza inicial já traz uma problematização: é criada a cátedra de “pediatria”, mas, como Sanglard e Ferreira (2010) apontaram, o primeiro catedrático é Barata Ribeiro, um higienista. Os autores indicam ainda que somente em 1928 – o que coincide com o recorte final por nós proposto – a FMRJ teria um pediatra à frente da cátedra, com a aprovação de Luiz Barbosa no concurso. Ao pretender pensar nos caminhos da saúde da criança na virada do século XIX para o XX dialogaremos diretamente com esse trabalho, revendo algumas afirmativas de ambos e avançando na análise acerca do processo de institucionalização da pediatria na cidade do Rio de Janeiro, então capital da República.

Para tal, vamos discutir as diferenças entre a “higiene infantil” e a “pediatria” na época, bem como tal questão aparecia nos congressos internacionais de assistência infantil; a seguir, analisaremos a relação entre Fernandes Figueira e a higiene infantil e, por fim, o papel da alimentação infantil na ação desse profissional.

## A institucionalização da pediatria no contexto internacional

Este tópico discutirá como, internacionalmente, a higiene infantil e a pediatria foram se constituindo no último quartel do século XIX, quando a puericultura (1863) e a pediatria (1872) surgem como especialidades autônomas. E, dez anos depois, ocorre, em 1883, o primeiro congresso internacional dedicado à proteção da criança. Foi em meio aos índices alarmantes da mortalidade infantil que se realizaram os debates voltados para a infância e que a preocupação com esse problema sanitário e demográfico ganhou espaço internacional (Rollet, 2001).

Para entender as propostas levadas a cabo por Fernandes Figueira nas décadas iniciais do século XX, é preciso compreender como se deu a institucionalização da pediatria no mundo ocidental. O século XIX pode ser definido na Europa, em termos médicos, pelo surgimento das especialidades médicas, pelo higienismo, pelo pasteurianismo e pela filantropia. Ao falar sobre a puericultura, saber médico surgido na França na segunda metade do século XIX, o jurista Ataulfo de Paiva afirma que esse saber é fruto da ciência médico-social, mas, sobretudo, fruto dos avanços da ginecologia, que reduziu a mortalidade e a morbidade das parturientes, permitindo ao médico “a oportunidade de concentrar a sua atenção na pessoa do recém-nascido” (Prefácio..., 1922, p.13).^1^ Paiva atribui ao médico francês Pierre Budin o desenvolvimento dessa especialidade, para quem a mortalidade infantil nada mais era do que falta de conhecimento da mãe. Então, cabia à medicina ensiná-la e dar-lhe “uma direção médica que a fizesse compreender a verdadeira inteligência de sua missão” (p.13). Esse é, sem dúvida o princípio que norteará diversas instituições de assistência à infância em todo o mundo.

Olivier Faure (1993) afirma que as descobertas de Pasteur abrem espaço para uma nova era: a da prevenção pela higiene, que viverá, na França, seu auge a partir de 1870. Mesmo que o medo da doença ainda esteja presente, a necessidade de combater os efeitos da doença – a morte – fará com que a preocupação com a prevenção, ou o que o autor chama de “pausteurianismo preventivo” (Faure, 1994), ganhe a agenda de políticos e filantropos. Para Faure (2009, p.54), o higienismo é fruto “de uma visão global do indivíduo” e por meio dele é que se “dá origem à saúde pública, que integra o indivíduo a um conjunto mais vasto, a sociedade, e dá a esta a prioridade sobre aquele”. Nesse discurso, a “doença” perde espaço para a “saúde”.

Esses dois pontos balizam o argumento principal deste artigo: a partir das discussões sobre higiene, com seu caráter preventivo, sua atuação no ordenamento da cidade e no corpo humano, vemos a criação de uma ciência – a higiene infantil – que terá no combate à mortalidade infantil sua principal atuação. Nela, a doença da infância será secundarizada, em face da possibilidade de evitá-la. Como se verá ao longo do artigo, esse será o caminho vitorioso no que tange à assistência à infância no Rio de Janeiro até 1928.

Outra característica do higienismo é a ampliação de sua atuação por meio de uma “proliferação impressionante dos manuais de higiene, associados ao desenvolvimento de uma literatura de cunho filantrópico, especializada em aliar os benefícios da limpeza corporal à boa educação” (Sant’Anna, 2007, p.212). No que tange à infância, a autora ressalta que a ideia dos micróbios aumentava o medo da “degenerescência da nação, devido ao enfraquecimento da infância” (p.218) e, sobretudo, com relação à alta mortalidade infantil. Esse é um dos aspectos que os médicos que se dedicam à infância vão considerar. Como se verá, para Fernandes Figueira (set. 1913), a prevenção se dará pela alimentação adequada, mas esse médico também levará em consideração as condições de moradia das famílias.

Conforme apontado, tão logo surgem as especialidades médicas voltadas para a infância, é realizado, em Paris, o primeiro Congresso de Proteção à Infância (Rollet, 2001). As discussões desse congresso se organizaram em torno de cinco comissões, cada qual com seu respectivo tema, a saber: (1) primeira infância; (2) infância abandonada; (3) aprendizes; (4) refratários da escola; (5) jovens detentos. Esses temas marcam os principais caminhos da assistência à infância: a primeira infância e a mortalidade infantil; e a delinquência infantil.

Na leitura dos anais desse congresso percebem-se os debates que se seguiram sobre a infância abandonada, tema de trabalho da segunda comissão; a higiene infantil foi acionada como meio para conquistar a saúde em sua integralidade. Nesse caso, vale ressaltar a fala do doutor Ladame (1885), diretor de um orfanato na Suíça que, ao discorrer sobre a criança abandonada, deixa claro o papel da higiene infantil naquele momento. Para ele, a “boa higiene” não deveria deixar de lado a história hereditária, de maneira a compreender as disposições nativas que deveriam ser combatidas ou encorajadas.

Ladame se ampara nos manuais de higiene do doutor Fonssagrives, da Faculdade de Medicina de Montpellier, para afirmar que a higiene infantil poderia subverter as consequências danosas da má hereditariedade. Como trata Gondra (2003), os manuais do higienista francês – escritos a partir de 1870 – foram relevantes no sentido de tornar o campo reconhecido como o mais apropriado para dirigir comportamentos tanto privados quanto públicos e legitimar-se como ferramenta para a formação integral do homem. Nesse sentido, a higiene atravessava as diferentes esferas e, no caso do manual do doutor Fonssagrives, tornava-se relevante para as educações física, moral e intelectual do indivíduo, para as quais o concurso “perseverante e simultâneo” dos pais e mães tornava-se imprescindível (p.32).

A higiene responderia aos problemas individuais e coletivos em sua integralidade, abarcando diferentes aspectos da vida social. Seria responsável por curar e assegurar o bem-estar de uma categoria de crianças – as abandonadas – que, segundo membros da comissão, estariam condenadas à fatalidade. Nesse sentido, os significados que a higiene ganhava no congresso aparentam semelhança com a definição presente no trabalho de José Gonçalves Gondra (2003, p.28), uma vez que mostrava ser uma ciência “compósita, resultante da aplicação de diversas ciências e com um único objetivo: o estudo das causas capazes de modificar a saúde e os meios capazes de anular ou diminuir a ação mais ou menos nociva das referidas causas”.

A higiene era um princípio a ser seguido em nome de um modelo de homem civilizado e que, na configuração da ordem médica, reivindicará para si a condição de ramo principal (Gondra, 2003). A pediatria, por outro lado, especialidade da medicina voltada para a saúde da criança, ainda estava em processo de consolidação e, portanto, não aparece de maneira bem delineada nos debates desse primeiro congresso. As discussões que se seguiram e que são transversais ao tema da saúde infantil giraram em torno dos hospitais infantis e da necessidade de sua fundação nas cidades por motivos de ordem moral e higiênica – notadamente a questão dos isolamentos. Além disso, entendendo que o destino da mãe estava intimamente ligado ao do filho, discutia-se a necessária multiplicação de dispensários infantis.

No primeiro Congresso Internacional pela Proteção da Infância, realizado em Florença, em 1896, a pediatria já aparece como alvo de debate. O professor florentino Pietro Celoni, relator da décima tese, que versava sobre as necessidades de clínicas e hospitais pediátricos e os meios para os conseguir, faz uma correlação direta entre a fundação da primeira clínica pediátrica e o primeiro hospital infantil, inaugurado em 1802, na França: o Hôpital des Enfants Malades. Segundo o médico, após a fundação do primeiro grande hospital dedicado à infância em Paris, era inaugurado o Hospital Infantil Nicolò, de São Petersburgo (1834), e, três anos depois, o St. Anna Kinderspital, em Viena. A partir de então acontece rapidamente a fundação de hospitais para crianças na maioria das cidades da Europa, e, com eles, a de clínicas majoritariamente ligadas aos hospitais ou mesmo independentes deles (Celoni, 1899, p.302).

De todos os hospitais citados, Celoni elenca o hospital de Moscou St. Vladimir, fundado em 1876, como modelo a ser seguido, sobretudo em se tratando de sua clínica. Nele a clínica estava conectada ao escritório de admissão e dispunha de uma sala de isolamento junto à sala destinada para casos suspeitos de contágio, uma sala de espera, outra que dava acesso às quatro salas de consultas para exames laringoscópios, oftalmoscópios etc. Uma sala nos fundos era destinada às consultas que demandavam cirurgias, e, além dessa, havia outra com quatro leitos para descanso de crianças que vinham de longe, que haviam sido operadas ou tinham saído do banho. Finalmente, um banheiro, bem como uma pequena farmácia, atendiam aos enfermos da clínica.

Para o médico florentino, as clínicas seriam de grande valia para as crianças que não necessitavam de internação, sendo tratadas sem as separar do seio da família e da escola. Serviriam também para dar lugar àquelas para as quais o hospital era urgente e, nesse sentido, diminuir os custos de internação que, segundo ele, seriam onerosos para todos os municípios. Além disso, as clínicas serviriam à ciência e ao ensino, proporcionando a oportunidade do estudo e fornecendo aos alunos os casos mais comuns e, portanto, mais relevantes para a prática médica, que de outra forma escapariam à observação e ao estudo (Celoni, 1899).

A importância de clínicas e hospitais infantis para o aprendizado das doenças da infância fez com que pediatras italianos reclamassem, ainda na década de 1890, a criação de tais estabelecimentos. Os médicos insistiam não só na necessidade de fundar hospitais infantis que tivessem autonomia em relação aos demais, mas na fundação de cadeiras universitárias direcionadas para as enfermidades da infância. A luta era em prol de hospitais infantis completos, equipados para assistir crianças desde a primeira idade, pois entendia-se que era nas doenças do lactente e em suas doenças infecciosas que o jovem médico se encontrava em déficit, tendo em vista que, durante as aulas, ele quase nunca tinha acesso a esses casos para os estudar (Mandelli, 1899, p.240).

Nesse sentido, ao longo do século XIX, o estudo da patologia infantil vai se autonomizando em estabelecimentos especiais. Esse processo, entretanto, encontra embargos dentro do grupo de médicos pertencentes às enfermarias dos grandes hospitais. O médico e presidente do Hospital Infantil de Cremona, Alfonso Mandelli (1899, p.241), que colaborou com a construção dos hospitais infantis de Milão, Veneza, Placência e Verona, informa que os obstáculos se deram na forma de publicações destinadas a provar a inutilidade dessas novas instituições diante da existência, nessas cidades, de hospitais com alas infantis.

Ao que parece, as dissonâncias dentro do campo giram em torno de um entendimento generalista da atuação médica, segundo o qual a formação pretensamente abrangente e a existência de hospitais com enfermarias infantis eram vistas como suficientes para assegurar que as crianças fossem bem assistidas. Além disso, criar especializações médicas implicaria, em algum nível, a perda de espaço de atuação para tais médicos.

Em meio à discussão promovida pelo médico bolonhês Giovanni Berti, ainda nesse congresso, Celoni faz um adendo a ser acrescentado em votação estabelecendo a presença de um especialista em pediatria ao lado do médico comum em todos os institutos onde a infância fosse recolhida. Talvez prevendo as prováveis oposições, trata logo em seguida de comunicar que a medida não visava substituir os médicos comuns, mas serviria para os pediatras prestarem os seus serviços nos casos em que isso fosse absolutamente necessário.

Tais embates podem ser percebidos em função da comunicação feita pelo médico Alfonso Mandelli, relator pela Itália da décima tese, junto com o professor Celoni. No seu parecer defende, sobretudo, dois pontos que serão alvo de debate e votação: (1) que as doenças das crianças apresentam peculiaridades no que diz respeito aos sintomas e tratamentos e que só podem ser reconhecidas e curadas a tempo por um médico dedicado ao seu estudo, ou seja, um pediatra; (2) que, por razões científicas, administrativas e morais, uma vez que as crianças doentes têm de ser separadas dos adultos doentes, existe a necessidade de hospitais pediátricos, que são também indispensáveis para o estudo e ensino das doenças infantis.

Sobre a primeiro ponto, o professor e médico napolitano Clemente Romano pede uma alteração. Segundo ele, a frase dá a entender que o tratamento de crianças estaria reservado exclusivamente aos pediatras, e que, portanto, nenhum outro médico poderia conhecer e tratar bem e a tempo essas doenças se não tiver cultivado o aprendizado das doenças das crianças (Primo Congresso..., 1899, p.245), o que demonstra que a pediatria ainda estava se firmando como saber autônomo.

Esse congresso foi dividido em cinco seções: (1) iniciativa de propaganda geral em favor das crianças; (2) melhoria física da infância e pediatria; (3) melhoria moral da infância; (4) melhoramento intelectual da infância; (5) questões econômicas. Ao longo dessas seções, alguns temas foram mais presentes e nos ajudam a entender a problemática que preocupava os médicos dedicados à saúde da criança.

Os temas que ganharam maior evidência foram, em primeiro lugar, a higiene, com 14 ocorrências; seguido da pediatria (nove) e ainda hospital infantil e idiotia (três cada um). Como se verá adiante, são temas que estarão diretamente envolvidos com a prática médica de Fernandes Figueira.

Okesi Otovo (2016) ressalta a participação de médicos brasileiros nos congressos internacionais de puericultura e assistência, adaptando as ideias neles discutidas à realidade local. A autora indica o caráter multicultural que a medicina brasileira irá ter a partir da década de 1920. Ainda segundo Otovo, essas conferências eram espaços para difusão do conhecimento da saúde materno-infantil, e os médicos brasileiros “participavam como representantes de toda a nação” (p.120).

As participações brasileiras, iniciadas em 1883, foram muito variadas, com a presença de médicos e delegados oficiais. Entre os médicos citamos Arthur Moncorvo de Figueiredo (DF) e João Teixeira Alves (SP). Moncorvo de Figueiredo, além de fazer parte do comitê executivo do primeiro Congresso Internacional de Proteção da Infância, de 1896, também integrou sua comissão internacional, sendo o único das Américas a participar como tal. Gradativamente, a presença de médicos brasileiros vai se firmando nos congressos internacionais de pediatria, puericultura, higiene e assistência.

A leitura das atas desses dois primeiros congressos mostra como a discussão sobre a saúde da criança estava se consolidando. A presença de médicos e delegados brasileiros era uma das formas com que essas ideias chegavam ao país, sobretudo a partir da publicação de artigos nos periódicos científicos mais importantes como *União Médica* (substituído depois pelo *Brasil Médico*) e pelo *Boletim* (depois, *Anais*) *da Academia Nacional de Medicina*.

A institucionalização da pediatria é um longo processo e foi acompanhado de perto pelos médicos brasileiros, sobretudo Carlos Arthur Moncorvo de Figueiredo (Moreira, 2020). Sobre uma disputa de espaço entre a higiene e a doença, Júnia Pereira (2008) afirma, seguindo Martagão Gesteira, que a pediatria teria nascido de “forma diferente em relação às demais especialidades”, ao entender que atuaria sobre todas as necessidades da criança, e não sobre um órgão ou doença específica. A autora ainda aponta que a controvérsia entre a pediatria e a puericultura estava baseada na diferença entre o “natural” e o “científico”, e é “na demarcação destes limites ... que os pediatras tornaram exclusivo o atendimento da criança doente” (p.85); enquanto a puericultura estava ligada a “uma concepção de prevenção da saúde” (p.84). Mais do que essa controvérsia, o que os congressos internacionais apontam é a preocupação dos médicos com a “higiene da criança”.

Sobre essa disputa no campo, o médico e pediatra carioca Luiz Barbosa (1914, p.105) afirma, em discurso à FMRJ publicado no *Brasil Médico*, que “as discordâncias frequentes e particularidades flagrantes que obrigam destoarem, por vezes, a semiologia e a patologia, quando conformadas em certas fases da vida”.^2^ Tal oposição será importante para compreendermos a prática médica de Fernandes Figueira.

Entenderemos a higiene infantil como o meio do caminho entre a puericultura e a pediatria. Se distancia da primeira, por ser liderada por médicos – enquanto a puericultura estava sendo criticada por poder ser exercida por não médicos; e da segunda, por não ter a doença como foco, mas a sua prevenção. No momento de definição do espaço de atuação da medicina da infância, demarcar o espaço de atuação era fundamental para esses homens.

Como se viu, aos poucos a pediatria vai ganhando espaço nas discussões, mas a higiene infantil ainda é muito forte, por seu caráter preventivo, notadamente no que se refere às necessidades da redução da alta mortalidade infantil – uma característica da virada do século XIX para o XX e fortemente relacionada ao pauperismo urbano ou às más condições de trabalho, salário e alimentação das classes populares nas grandes cidades. A alimentação infantil, sobretudo da criança até 1 ano, será um dos pilares da higiene infantil – não à toa esses médicos serão grandes defensores do aleitamento materno e combaterão o uso indiscriminado das amas de leite, deixando-as exclusivamente para os casos em que as mães não podiam aleitar seus próprios filhos por óbito ou doença.

Esse é o cenário em que o ensino de pediatria será institucionalizado no país (1883) e no qual Antônio Fernandes Figueira irá se formar (1887) e iniciar sua carreira profissional.

## Fernandes Figueira e a higiene infantil

É por meio da produção de manuais de vulgarização científica e da participação nos congressos internacionais que as ideias da higiene infantil chegam ao Brasil do século XIX. Em 1859, o médico e professor da FMRJ Antonio Ferreira Pinto publica seu livro de vulgarização, fortemente inspirado na publicação de E. Bouchut, *Tratado prático das moléstias dos recém-nascidos e das crianças de peito*, publicado na França, em 1855 – e também nos trabalhos de Velpeau, Caseaux, Monneret e Morel. Ressalta os cuidados com a infância, prega o aleitamento materno e responsabiliza o marido e senhor de escravo pela gravidez da esposa e da escrava, a quem dedica cuidados especiais (Pinto, 1859).

Sem dúvida, o mais importante dos projetos de vulgarização científica é o jornal *A mãe de família*, publicado initerruptamente no Rio de Janeiro entre 1877 e 1888 e que teve no médico da corte, Carlos Costa, sua principal liderança. Segundo Karoline Carula, *A mãe de família* surge a partir da coluna “Cartas às senhoras brasileiras”, publicada na *Gazeta de Notícias* em 1877. Se a publicação da coluna possibilita voos mais altos, como a criação de um periódico, tal empreitada ganha nova dimensão e alcance. Carlos Costa se inspira fortemente no jornal francês *La Jeune-Mère*, editado pelo também médico André Théodore Brochard. O jornal teve um longo alcance, sendo vendido na corte e nas províncias de Rio de Janeiro, Minas Gerais e São Paulo. Dos vários temas nele discutidos, Carula (2012) salienta o papel da alimentação infantil e a crítica do uso de amas de leite na criação das crianças.

A crítica ao uso de amas de leite não é novidade e está presente nos manuais de conselhos médicos ao menos desde o século XVIII – período em que começam a ser publicados os tratados médicos voltados para as crianças (Sá, 2011). Isabel dos Guimarães Sá (2011) ressalta que a criação externa e o uso de amas de leite eram práticas comuns entre a elite, que passou para burguesia e, no século XIX, em função do trabalho fabril, chegou às classes populares. No Brasil, as amas de leite eram majoritariamente escravas, contudo, os estudos de Caroline Gil (2018) apontam que, no Rio de Janeiro das primeiras décadas do século XX, a oferta e a procura de amas de leite explodiram na cidade, espraiando-se por todo o tecido urbano e social.

O jornal *A mãe de família* foi importante para a difusão de preceitos higienistas e abriu caminho para o longo processo de responsabilização da mulher no cuidado do bebê, criando a ideia de maternidade. Também se caracterizou por publicar ensinamentos de educação, economia doméstica e outros textos/colunas voltados para o papel da mulher no lar (Carula, 2013).

Carlos Costa, entretanto, como principal editor do periódico, não se dedicou apenas a esse veículo para divulgar os preceitos higienistas. Ministrou palestras sobre higiene na Escola Noturna de Botafogo, em 1877, voltada para a instrução da classe operária, falando a respeito de higiene corporal, da necessidade de realizar esportes aquáticos, das vestimentas e da alimentação, e salientava que era possível conciliar o bolso com a higiene (Carula, Freire, 2017). Interessava que seus alunos fossem propagadores dos preceitos higienistas.

Karoline Carula (2016) aponta que o incentivo ao aleitamento materno era tema recorrente nas teses da FMRJ, antes mesmo da criação da cátedra de pediatria. Assim, ao ser criada a cátedra, em 1883, as discussões sobre a higiene infantil já circulavam na cidade – tanto na vulgarização quanto nas discussões médicas. Fernandes Figueira entra na FMRJ em 1881, tendo se doutorado em 1887. Ele é aluno da FMRJ quando a cátedra é implementada.

Em artigo sobre a institucionalização da pediatria no Rio de Janeiro, Gisele Sanglard e Luiz Otávio Ferreira (2010) afirmam a liderança de Carlos Arthur Moncorvo de Figueiredo no processo de criação da cátedra na FMRJ e apontam que a aprovação do médico higienista Barata Ribeiro no concurso teria postergado a presença de um pediatra no ensino dessa especialidade – discurso presente entre os médicos-memorialistas, como aponta Júnia Pereira (2008). Deixando de lado a não participação de Moncorvo de Figueiredo no certame, explorada pelos autores, gostaríamos de nos centrar em dois aspectos: o primeiro, o fato de Barata Ribeiro ser um higienista que, como já visto, estava em consonância com as discussões da época, em que a preocupação com a saúde da criança e o caráter preventivo da higiene infantil se sobrepunham à doença da infância. O segundo ponto, a vinculação de Fernandes Figueira a Moncorvo de Figueiredo, como seu discípulo e aluno nos cursos livres de moléstias das crianças na Policlínica Geral do Rio de Janeiro.

Essa vinculação, repetida inúmeras vezes pela historiografia brasileira, vem sendo questionada por Gisele Sanglard desde 2016. A autora, ao reconstituir a trajetória de Fernandes Figueira, aponta sua proximidade com Barata Ribeiro, de quem ele foi assistente até 1900, quando lançou seu livro *Sémiologie infantile* e se notabilizou, nacional e internacionalmente, como pediatra (Sanglard, 2016), partindo para uma carreira pública importante como chefe dos serviços voltados para a infância do Hospital São Sebastião e do Hospício Nacional de Alienados. É o próprio médico que se define como discípulo do higienista quando, em virtude de uma palestra feita na Policlínica das Crianças, em 1914, se refere a Barata Ribeiro como seu mestre ao falar sobre a sífilis na infância: “Muito antes dos trabalhos fundamentais de Hochsinger, Barata, meu mestre, inculpava a sífilis na produção do mixedema, e tenho notícia de uma cura nesse sentido” (Figueira, 1914, p.103).

A cátedra de pediatria na FMRJ, portanto, caracterizou-se, em seu início, com o viés da higiene infantil. De 1883 a 1910, ano de morte de Cândido Barata Ribeiro, os médicos por ele formados trabalhavam na lógica da saúde da criança e com a higiene infantil. Da turma de 1887 fizeram parte Fernandes Figueira e Olinto de Oliveira; um seguiu pela saúde da criança e a higiene infantil; o outro foi discípulo de Moncorvo de Figueiredo, que se notabilizou pelas doenças da infância.

O discurso médico acerca da história da pediatria, conforme Júnia Pereira (2008) ressaltou, constrói uma linha teleológica a partir Moncorvo de Figueiredo. Médicos como Martiniano da Rocha e Walter Telles afirmam que a especialidade só se efetivaria de fato no Rio de Janeiro em 1895 – excluindo qualquer divergência e reificando Moncorvo de Figueiredo no papel de “pai da pediatria”, discurso que será recuperado por historiadores (Sanglard, Ferreira, 2010; Moreira, 2020). Segundo Júnia Pereira (2008, p.71), W. Telles afirma que Barata Ribeiro, o primeiro catedrático de Pediatria da FMRJ,

relegaria a cátedra por longa data para se dedicar primeiramente à propaganda republicana e, em seguida, para ocupar sucessivamente os cargos de presidente do Conselho Municipal, prefeito do Distrito Federal e senador pelo Distrito Federal, chegando a ser indicado para o Supremo Tribunal Federal.

Sua militância política teria feito com que a cátedra não tivesse funcionado como se esperava. Contudo, como apontado, Fernandes Figueira se apresenta como discípulo do médico e político. Certamente não terá sido o único, mas indubitavelmente o mais famoso deles.^3^

Ressaltamos que a marca da higiene infantil esteve presente na cátedra da FMRJ: em 1883 foi criada a “clínica e policlínica médica e cirúrgica de crianças”, que, com a morte de Barata Ribeiro, em 1910, foi desdobrada, no ano seguinte, em duas cadeiras: “clínica de pediatria médica e higiene infantil” e “clínica cirúrgica infantil e ortopédica” (Sanglard, Ferreira, 2010). Já o curso livre da Policlínica Geral do Rio de Janeiro, criado e dirigido por Moncorvo de Figueiredo, era de “moléstia das crianças”.

Os temas caros à higiene infantil e as temáticas discutidas no congresso de 1896 serão facilmente percebidos na atuação de Fernandes Figueira. Uma das grandes preocupações do médico era com relação à separação de adultos e crianças nos hospitais, bem como à presença das mães acompanhando seus filhos. Suas primeiras ações nesse sentido serão no Hospital São Sebastião, hospital de isolamento da prefeitura do Distrito Federal onde começará a trabalhar em 1902 (Sanglard, 2008).

Sobre a criação de hospitais infantis, ressaltamos a da Policlínica das Crianças Pobres da Santa Casa da Misericórdia do Rio de Janeiro, em 1909. Sua construção se deu graças à benemerência de José Carlos Rodrigues, então diretor do *Jornal do Commercio*, e irmão da pia instituição. Fortemente inspirada na experiência do Hôpital des Enfants Malades, dirigido por Victor Hutinel na capital francesa, a Policlínica das Crianças teve como seu diretor Fernandes Figueira, a quem coube dar-lhe o desenho técnico. Espaço sobretudo ambulatorial, foi nas dependências da instituição que as políticas públicas de Fernandes Figueira começaram a ser desenhadas e onde ele formou sua Escola de Pediatria ou de higiene infantil, tendo sido espaço da prática médica da FMRJ até a criação do Hospital São Zaccharias, em 1914, a despeito de ele não ter sido jamais professor da instituição (Sanglard, 2014, 2016; Sanglard, Ferreira, 2010, 2014).

Fernandes Figueira também teve longa atuação no Hospício Nacional de Alienados, onde foi o responsável por organizar e dirigir o pavilhão Bourneville (1903), voltado para as crianças alienadas – sobretudo para aquelas diagnosticadas como “idiotas”. A idiotia era doença que preocupava os higienistas e era fartamente discutida nos congressos médicos, como o de 1896 em Florença. O tratamento proposto pelo médico francês Désiré-Magnolle Bourneville possibilitava inserir o idiota no mercado de trabalho, ensinando-lhe ofícios (Roma, Sanglard, Muñoz, 2022). Fernandes Figueira divergia do método de Bourneville no que tange à finalidade do tratamento, pois, para ele, “o idiota estará fadado a perpétua internação, e sua manutenção teria que ser garantida pelo seu trabalho na instituição” (p.138).

A maior parte das crianças no Pavilhão Bourneville era pobre, público-alvo do médico brasileiro (Sanglard, Ferreira, 2014). Com relação à criança idiota, o método “foi utilizado não apenas para tornar a criança anormal produtiva, mas também como meio de prevenção de um futuro ônus econômico e social ao país” (Roma, Sanglard, Muñoz, 2022, p.139).

Se o alcance do pavilhão Bourneville era limitado (Roma, Sanglard, Muñoz, 2022), foi no combate à mortalidade infantil que a política de higiene infantil de Fernandes Figueira se notabilizou.

## A alimentação infantil no cerne do combate à mortalidade infantil

O combate à mortalidade infantil ganhou evidência no Brasil na virada do século XIX para o século XX, muito carreado pela preocupação demográfica. Essa discussão já estava presente entre os higienistas europeus, conforme pode ser percebido nos congressos de assistência à infância aqui apresentados. A presença do escravismo, como garantia de mão de obra, adiou a chegada dessa discussão no país. A promulgação da Lei do Ventre Livre, em 1871, trouxe o problema da reposição da mão de obra, e é nesse contexto que devemos entender a preocupação com a mortalidade infantil.

O anuário estatístico de 1890 dá uma pista da dimensão que a mortalidade infantil toma na cidade do Rio de Janeiro. Aureliano Portugal, chefe da Diretoria de Estatística do Distrito Federal, ressalta que o crescimento populacional só não é vegetativo graças à imigração. O médico afirma que a taxa de mortalidade infantil (de 0 a 1 ano) representa 18,4% da totalidade de mortes em geral – excetuando os períodos epidêmicos. Frisa que esses números não expressam a realidade, conforme os higienistas brasileiros já indicavam – o grande problema, segundo ele, era a baixa natalidade na cidade, sempre inferior à mortalidade. E, continua, se há um aumento da população adulta, deve-se exclusivamente à imigração. Com essa explicação, ele apresenta o número alarmante da mortalidade dessa faixa etária: 294,8% em 1872 e 213,6% para 1890 – a diminuição é justificada pela redução da mortalidade geral na cidade (Portugal, 1891).

Não à toa o tema da mortalidade infantil, sobretudo da primeira infância, animou médicos e filantropos brasileiros no período, caracterizado pela abertura de diversas instituições dedicadas à assistência à infância: em 1899 foram criados o Instituto de Proteção e Assistência à Infância, por Moncorvo Filho, e a Policlínica de Botafogo, por Luiz Barbosa, que teve no serviço de pediatria um de seus principais serviços; em 1909 foi inaugurada a Policlínica das Crianças Pobres da Santa Casa da Misericórdia do Rio de Janeiro (SCMRJ), como comentado; em 1914, o Hospital São Zaccharias, da SCMRJ, espaço da prática médica da cátedra de pediatria da FMRJ. Por fim, em 1924 foi inaugurado o Hospital Abrigo Arthur Bernardes, ligado à IHI, que sintetiza a política de assistência à infância de Fernandes Figueira.

Os dados relativos ao peso da criança e suas flutuações poderiam ser indicativos de uma alimentação deficitária – tema que será abordado por ele e por seus alunos. Os riscos de uma hiperalimentação também serão ressaltados pelo médico que, para evitar tal situação, recomendava o intervalo de três a três horas e meia entre as refeições, totalizando seis refeições diárias.

Fernandes Figueira fazia um balizamento entre as escolas francesa e alemã de pediatria. Como exemplo podemos ressaltar a última questão levantada: para os médicos franceses, as crianças da primeira infância deviam ser alimentadas sete a oito vezes ao dia, e o uso de mingaus era tardio; já na escola alemã, eram cinco refeições ao dia. Fernandes Figueira indicava que as crianças deveriam ser aleitadas seis vezes ao dia, com intervalos regulares, e, em casos excepcionais, podiam ter sete mamadas. Sobre os mingaus, indicava a primeira oferta a partir dos seis meses (Figueira, 1919).

A alimentação infantil será um dos principais aspectos de intervenção de Fernandes Figueira e da escola de pediatria que criará ao redor de si. Para garantir crianças saudáveis, adotar-se-iam medidas preventivas que evitassem o seu desequilíbrio fisiológico. Os serviços de higiene infantil, cuja atuação se dava no sentido de evitar as potenciais doenças, serão dos mais relevantes na concepção do médico. No cerne de sua política, a defesa intransigente do aleitamento materno.

Segundo ele, a higiene infantil era assunto prioritário do pediatra, uma vez que considerava que ali estava a base essencial da clínica infantil (Figueira, 1914, p.109). A inserção da higiene se dava no sentido de evitar as moléstias infantis, e não necessariamente tratá-las. Assim, encaixava-se em seu escopo uma gama bastante abrangente de ações e prescrições: desde aquelas voltadas para a melhoria das condições habitacionais da população, passando por uma legislação protetiva da mãe trabalhadora e de seu filho, até a questão das criadeiras, da educação, da alimentação infantil entre outras.

Considerava que o “rumo verdadeiro” a ser dado à cadeira de clínica infantil no ensino médico passaria pela criação de uma outra, de “higiene e clínica da primeira infância”, de modo a abranger de “maneira mais condigna” os assuntos médicos que envolviam a infância (Figueira, 1913, p.100). Para ele, tal necessidade se justificava pela situação de vida precária das crianças na cidade:

Não há dúvida [de] que em país como o nosso se figure imperativo o estudo científico e popular da higiene da primeira infância. Habitamos uma cidade de alimentação péssima, vida fatigante e abandono criminoso da população infantil. Excetuando os benefícios ministrados pela magnânima classe médica, pelos estabelecimentos da Santa Casa, pelo Instituto Moncorvo e pouco mais, da lei nada recebe aqui a criancinha (Figueira, 1913, p.101).

Pouco antes de publicar esse artigo, Fernandes Figueira havia publicado, em 1910, *O livro das mães*, que teve duas outras edições (1919 e 1926), com pequenas alterações. Escrito de forma epistolar, cada “capítulo” responde a uma pergunta de um remetente desconhecido. Por meio dessas cartas, Fernandes Figueira trata dos principais temas sobre a saúde da criança de até 1 ano: setenta das 107 consultas versam sobre a alimentação infantil, ou seja, 65,42%. A preocupação com a dietética infantil é característica da escola francesa, para a qual tal questão se “relacionava à imunidade adquirida na alimentação com a prevenção de doenças” (Loch, 2020, p.339).

Para o médico, a alimentação impactaria o desenvolvimento da criança. Todos os problemas passavam pela alimentação: reduz o óbito entre os prematuros e mantém o lactante saudável. Das setenta consultas dedicadas ao tema da alimentação infantil, 30% versam sobre o leite de vaca, das quais três são relativas ao leite condensado; 70% sobre aleitamento materno, mingaus, farinhas e amas. Nessa discussão, critica as “Gotas de Leite”,^4^ que para ele induzem a outro grave problema: o sobrepeso.

A alimentação artificial era dividida em dois grupos: os leites e os alimentos industrializados. Do primeiro grupo sobressai o leite de vaca, por ser mais acessível. Indica a inadequação à criança com menos de 5 meses, por causar distúrbios gástricos – maior índice de óbito nessa faixa etária. Para crianças menores de 5 meses indica usar leite desnatado, ensinando às mães como realizar a tarefa em casa.

Também levanta preocupação com relação à qualidade do produto, instigando as mães a ter cuidado com a saúde das vacas; com o asseio dos vaqueiros; do transporte (os vasilhames); devem procurar saber como se deu o resfriamento do produto – uma vez que a qualidade dos estábulos no Rio de Janeiro era questionável, e o produto vindo de “Minas” (montanhas de Minas Gerais, Rio de Janeiro e São Paulo) era um pouco melhor. Avultam impurezas no leite proveniente dos estábulos do Rio de Janeiro, e no das montanhas, o leite é irregularmente resfriado (Figueira, 1919). A questão do controle do leite na cidade do Rio de Janeiro nesse período foi amplamente explorada por Caroline Gil (2022) em sua tese. Figueira não se restringe à crítica do produto: ensina o processo de tratamento doméstico do leite.

Com relação aos produtos industrializados disponíveis, o médico aponta para o baixo custo do leite condensado, que, por ser desengordurado, não causa diarreia nas crianças. Comenta o esforço na sua importação, mas o indica apenas para viagens.

Considera os produtos maltados mais benéficos, mas fala da necessidade de a indústria brasileira produzir extrato de malte, uma vez que, por ser produto importado (Uruguai, Argentina e EUA), se torna inacessível aos pobres – para quem ele considera mais indicado.

Com relação às farinhas, afirma que a farinha láctea é a única indicada, por ter na sua composição leite condensado suíço, pão de trigo e outros cerais. Já as outras farinhas disponíveis para compra podem ser facilmente substituídas por preparados domésticos com base em maisena, araruta ou farinha de arroz, desde que misturados ao leite desengordurado.

A preocupação com o sistema digestivo da criança pautou a preocupação do médico; entende-se sua luta pelo aleitamento materno e outras fórmulas que o não afetam. No *Livro das mães*, Fernandes Figueira (1919) já indica que, a partir dos 8 meses, a criança já pode começar a ingerir algumas papinhas. Sua preocupação é vista nas teses defendidas na FMRJ pelos alunos por ele orientados (Sanglard, 2016). Um bom exemplo é a tese de Mario Gomes defendida em 1916, sob sua orientação. Nela, o jovem médico realiza um inquérito com 130 crianças que moram nas redondezas da Policlínica das Crianças – na região central da cidade.

**Figura 1:**
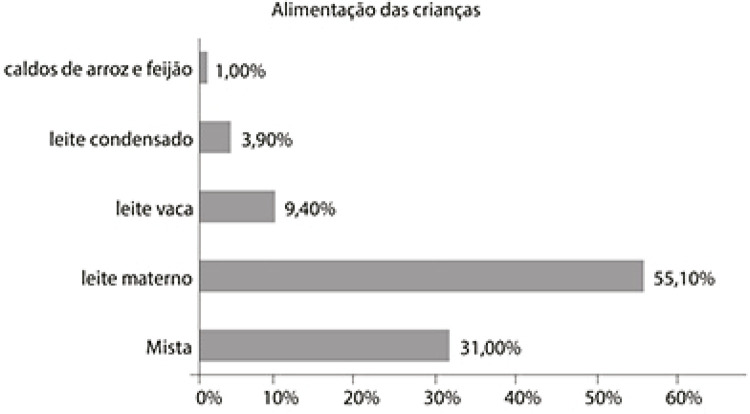
: Alimentação das crianças (elaborada por Gisele Sanglard com base na tese de Mario Gomes)

Sanglard e Ferreira (2014) apontam que a maior presença do aleitamento materno indica que a atuação de Fernandes Figueira nessa região pauperizada da cidade tinha tido algum alcance. A alimentação mista é bastante diversificada, podendo ser leite materno e leite condensado; ou, ainda, ambos os leites e leite de vaca; leite materno, condensado e sopas/mingaus etc. Essas misturas, com base no leite materno, indicam, certamente, a condição de trabalhadora da mãe. O trabalho desse jovem médico reitera a preocupação de seu orientador com a alimentação do filho do trabalhador pobre; e a realização de inquéritos sociais.

N’*O livro das mães*, Fernandes Figueira defende a criação de espaços voltados para a amamentação materna próximos às fábricas: as câmaras de lactantes, tema já explorado em outro estudo (Sanglard, 2016). Outra sugestão de Figueira é copiar um exemplo que, segundo ele, estava sendo posto em prática na Europa, na França em particular: pagar as mães para aleitar seus filhos, compensando o que perdem nas fábricas – não há notícia de qualquer tentativa de colocar em prática tal proposta por aqui.

A preocupação com a mãe trabalhadora move a resolução do problema da infância por Fernandes Figueira: dar condições de elas aleitarem e combater o aleitamento mercenário (amas de leite). Sobre o uso das amas, conforme já explicitado, era restrito a dois momentos específicos. Negava a existência de “leite fraco”; para ele, a mulher poderia estar subnutrida e então não ter leite suficiente – o problema era da mulher, e não do leite. Outra questão que incomodava muito o médico era o que ele chamava de o “irritante” problema do Rio de Janeiro: a contratação da ama significava que seu filho seria cuidado por uma mulher mais pobre do que ela própria. O correto seria a ama aleitar seu próprio filho e aquele para o qual fora contratada; afinal, uma mulher poderia aleitar até três crianças sem prejuízo (Figueira, 1919).

Com relação à alimentação infantil, o médico foi um ferrenho crítico à proliferação de instituições que distribuíam leite esterilizado, a exemplo das Gotas de Leite; para ele, tais organizações incentivavam o aleitamento artificial. Também era crítico à noção de robustez, pois indicava hiperalimentação. Ressalte-se que estavam em voga, na cidade, os Concursos de Robustez capitaneados por Moncorvo Filho (Freire, Leony, 2011), que premiavam crianças “robustas”; para Fernandes Figueira (1919), robustez não era sinal de saúde. Esses eram alguns dos muitos pontos que opunham ambos os médicos que atuavam em prol da assistência à infância na cidade na época.

A partir da Policlínica das Crianças, Fernandes Figueira vai, gradativamente, transformando a assistência à infância e nucleando seus alunos. O primeiro é Antonio Santos Moreira, que fora seu assistente na Policlínica das Crianças e, a partir de 1910, se torna médico da creche da Casa dos Expostos da SCMRJ. No relatório publicado em 1912, Santos Moreira fala das melhorias realizadas no serviço a seu cargo e na diminuição da mortalidade entre as crianças até 1 ano (Casa dos Expostos, 1922; ver também a nota 1). Figueira atribui a isso os testes realizados por ele na Policlínica das Crianças com a sopa Keller – um extrato maltado amplamente utilizado por Santos Moreira na Casa dos Expostos (Figueira, 1919).

Conforme Gisele Sanglard (2016) indica, analisando o último livro organizado por Fernandes Figueira (1929), publicado postumamente, pode-se perceber a presença de médicos que compuseram a primeira geração da Policlínica das Crianças em diversos hospitais da cidade, bem como em diversos cargos no DNSP.

## Considerações finais

Para concluir, ressaltamos que Fernandes Figueira foi o pediatra convidado para construir as primeiras políticas públicas para a infância na reforma da saúde pública levada a cabo por Carlos Chagas. Conforme Gisele Sanglard e Luiz Otávio Ferreira (2014) indicam, Fernandes Figueira era próximo de Carlos Chagas e Oswaldo Cruz, e é essa proximidade que o leva para o DNSP e lhe permite estabelecer as linhas de ação da IHI.

Apesar das ações limitadas de atuação no Distrito Federal, os convênios do DNSP fizeram com que as ações da IHI chegassem a outros estados como Bahia e Pernambuco (Sanglard, 2016).

É na IHI que se veem as discussões levadas a cabo por Fernandes Figueira se transformarem em políticas públicas. Desde 1905, o médico defende a separação da atuação da filantropia da atuação pública. À primeira caberiam a abertura de creches e de escritórios de lactante, que, como ele mesmo diz, eram baratos, pois bastava uma balança e um microscópio, enquanto aos poderes públicos, todo o resto.

Nem tudo foram flores na liderança da IHI. Fernandes Figueira viu-se obrigado a criar um sistema de atestação de amas de leite público, mas deixou claro no *caput* da lei sua oposição ao sistema (Gil, 2018).

A Policlínica das Crianças foi laboratório das propostas de assistência à infância e se tornou o lugar onde boa parte das ações eram realizadas, como o curso de Puericultura proposto pela IHI e realizado em suas dependências (Sanglard, Ferreira, 2014).

O ponto alto da política de Fernandes Figueira foi, contudo, a criação do Hospital-Abrigo Arthur Bernardes, vinculado à IHI, em 1924, que contou com vários médicos treinados por ele na Policlínica das Crianças (Sanglard, 2016). O hospital dedicava-se à internação de crianças até 2 anos de idade com graves problemas digestivos ou distúrbios alimentares, além de contar com espaços de laboratório para exames biológicos e físico-químicos, solário para helioterapia, gabinete de fisioterapia e gabinete de exame radiológico. Outra marca da política de infância de Fernandes Figueira era a presença da creche, para atender às crianças em fase de amamentação. A sua cozinha era, segundo Luiz Otávio Ferreira e Lidiane Ribeiro (2016, p.21), “o principal laboratório do abrigo” e

funcionava com espaço de aprendizagem para mães das crianças que acompanhavam seus filhos internados e para moças matriculadas na escola maternal que lá receberiam instruções sobre como preparar alimentos corretos para crianças na fase da primeira infância.

Espaço de atendimento e aprendizado para as crianças e suas mães e lugar onde a alimentação da infância e sua saúde ganharam evidência e marcam, até hoje, as ações do Instituto Fernandes Figueira, da Fiocruz, como o hospital-abrigo passou a se chamar na década de 1940, em homenagem a seu idealizador.

Até 1928 a higiene infantil norteou as ações da assistência à infância. Em 1927, com a mudança no governo federal, Fernandes Figueira deixa a IHI, que passa a ser dirigida por Olinto de Oliveira, seu amigo, mas que tinha uma linha diferente daquela defendida por ele: pendia mais para as moléstias infantis.

A higiene infantil encontraria naquele que se autodenominou seu herdeiro intelectual, o médico baiano Martagão Gesteira, seu grande incentivador por meio da Liga Bahiana contra a Mortalidade Infantil e, posteriormente, do Instituto de Puericultura da Universidade do Brasil (Ribeiro, 2011, 2020). E a pediatria passaria a dominar a cátedra da FMRJ. Menos uma disputa de espaço, mais sinais das mudanças do tempo. O desaparecimento precoce de Fernandes Figueira sem dúvida contribuiu para a mudança no equilíbrio entre ambos os saberes, mas suas propostas continuaram por muito tempo ensinando gerações de pediatras na FMRJ, por meio de *sémiologie infantile* e, sobretudo, por meio da instituição por ele criada.

## Data Availability

Não estão em repositório.

## NOTAS

1 Ataulfo de Paiva foi convidado pelo então prefeito Serzedello Correia (1909-1910) para organizar a obra *Assistência pública e privada no Rio de Janeiro: história e estatística,* cujas informações coligidas referem-se a 1912-1913. Por alguma razão, a obra só foi lançada em 1922, no âmbito das comemorações do Centenário da Independência. O prefácio à edição já estava pronto ao menos em 1916, quando é publicado o livro *Justiça e assistência*, de autoria do jurista, e que permite que ele seja eleito para a Academia Brasileira de Letras (Paiva, 1916). Na obra de 1922 são republicados dois capítulos da coletânea de 1916: “Assistência pública e privada no Rio de Janeiro” e “Assistência metódica”. O primeiro ganha atualização, sobretudo para marcar os novos rumos que a assistência à infância irá tomar com a direção de Fernandes Figueira; o segundo é publicado na íntegra. Vale ressaltar que “Assistência metódica” é a tese defendida por Paiva em 1908, no primeiro Congresso Nacional de Assistência Pública e Privada, e que tinha sido reprovada.

2 Virlene Moreira (2020, p.481), em artigo sobre a trajetória de Moncorvo de Figueiredo, define a semiologia como “basilar na construção da pediatria, assinalava o quanto o corpo da criança era complexamente especial, pois ao mesmo tempo frágil e potencialmente em “desenvolvimento”, e “a cura exigiria algo mais que acompanhar o progresso (ou causa) das doenças, tendo a terapêutica que se adaptar à fase de cada corpo”.

3 Em memória apresentada à Academia Nacional de Medicina, Barata Ribeiro (1898, p.21), na análise do caso apresentado, cita três médicos que o acompanharam naquele caso específico: Fernandes Figueira, “ex-assistente de clínica e hoje distintíssimo clínico no estado de Minas Gerais”; o doutor Arlindo de Souza, “colega”; e o assistente de clínica Augusto de Freitas, “então interno do meu serviço clínico”.

4 As Gotas de Leite são instituições criadas na França, na segunda metade do século XIX, com o objetivo primordial de distribuir leite esterilizado às crianças, como forma de combate à mortalidade infantil e muitas vezes associadas ao atendimento ambulatorial das crianças atendidas. O modelo ganhou o mundo, com formas distintas de atuação (Birn, 2006, 2007; Freire, 2009).
